# ﻿Species of the snake eel genus *Yirrkala* Whitley, 1940 from Taiwan, with descriptions of a new species and two new records (Anguilliformes, Ophichthidae)

**DOI:** 10.3897/zookeys.1220.130885

**Published:** 2024-12-09

**Authors:** Yusuke Hibino, Hsuan-Ching Ho

**Affiliations:** 1 Kitakyushu Museum of Natural History and Human History, Fukuoka 805-0071, Japan Kitakyushu Museum of Natural History and Human History Fukuoka Japan; 2 Department and Graduate Institute of Aquaculture, National Kaohsiung University of Science and Technology, Kaohsiung 81157, Taiwan National Kaohsiung University of Science and Technology Kaohsiung Taiwan; 3 Australian Museum, Sydney 2010, Australia Australian Museum Sydney Australia

**Keywords:** Biodiversity, catadromous, freshwater, ichthyology, slender eel, taxonomy

## Abstract

The slender snake eel genus *Yirrkala* from Taiwan is reviewed, and a total of four species are recognized, including a new species described here. *Yirrkalankust***sp. nov.** is described based on four specimens collected from western Taiwan. It can be distinguished from congeners by the dorsal-fin origin situated above the gill opening, the tip of lower jaw not reaching the base of the anterior-nostril tube, 1 + 3 supraorbital pores, 7–8 predorsal vertebrae, and 147–152 total vertebrae. Two rare species, *Yirrkalakaupii* Bleeker, 1858 and *Yirrkalaomanensis* Norman, 1939, are redescribed based on specimens newly collected from Taiwan. A key to all *Yirrkala* species found in Taiwan is provided.

## ﻿Introduction

The ophichthid genus *Yirrkala* Whitley, 1940 is a rare genus characterized by the combination of the following features: body elongate, cylindrical; tail length generally equal or less than half of total length; dorsal-fin origin generally above or behind gill openings; no pectoral fins; snout subconical; anterior nostrils tubular; teeth conical, mostly uniserial; gill openings ventral; and two preopercular pores (McCosker et al. 2007; McCosker 2022).

The name *Yirrkala* is derived from the local name of northern Caledon Bay, the type locality of its type species, *Yirrkalachaselingi* Whitely, 1940. Members of the genus mostly inhabit shallow-water habitats less than 100 m deep, such as those found in estuaries, beaches, and coral reefs; some habitats are unknown but possibly shallow (McCosker 2006, 2011, 2022; Fricke et al. 2024). Two species, *Yirrkalagjellerupi* (Weber & de Beaufort, 1916) and *Yirrkalakaupii* (Bleeker, 1858), also live in freshwater in rivers, and these species are rarely observed in the field. This genus comprises 18 nominal species, but it requires further revision due to the complicated taxonomy and a lack of samples. For example, 12 valid species are known solely from their type localities as documented in the original descriptions (Fricke et al. 2024). In contrast, *Yirrkalalumbricoides* (Bleeker, 1864) and *Yirrkalamisolensis* (Günther, 1872) are known from broader areas, although only *Y.misolensis* has been mentioned frequently (McCosker et al. 2006; Ho et al. 2015; Motomura et al. 2017; Chiu et al. 2022).

In Taiwanese waters, only *Y.misolensis* was previously known (Ho et al. 2015; Chiu et al. 2022). Recent surveys in the Penghu Islands (Pescadores Islands) have revealed many rare snake eels, such as *Apterichtushatookai* Hibino, Shibata & Kimura, 2015, *Callechelyskuro* Kuroda, 1947, and several unknown *Yirrkala* specimens. Others were collected from river mouths off Hualien by the aboriginal people using traps or fyke nets. In addition, at fish-landing port of southwestern Taiwan YH also found a fresh individual of *Yirrkala* which was identified as a species described by Norman (1939). Here, we describe a new species and redescribe two rare species; all three are newly recorded from Taiwan.

## ﻿Materials and methods

Methods for taking measurements and counts and terminology generally follow McCosker (2011). Measurements for total and tail lengths were taken by 300 or 600 mm rulers and others by digital calipers to the nearest 0.1 mm. Vertebral counts were made from x-ray photographs. Mean vertebral formula (**MVF**) is expressed as the average of predorsal, preanal, and total vertebrae. Total length is abbreviated as **TL**. All materials here we used are deposited in the Natural History Museum, London, UK (**BMNH**), the California Academy of Science, San Francisco, USA (**CAS**), the Kitakyushu Museum of Natural History and Human History, Kitakyushu, Fukuoka, Japan (**KMNH VR**), and the fish collection of the National Museum of Marine Biology and Aquarium, Pingtung, Taiwan (**NMMB-P**).

## ﻿Results

**Family Ophichthidae**

### ﻿Genus Yirrkala

Taxon classificationAnimaliaAnguilliformesOphichthidae

Whitley, 1940

5FE3D06F-C08D-5749-B35B-4BF09A9C8A55

Yirrkala Whitley, 1940: 410 (type species: Yirrkalachaselingi Whitley, 1940, by original designation).

#### Distinguishing features.

Body elongate, cylindrical; tail length generally equal or less than half of total length; all fins low but visible, dorsal-fin origin generally above or behind gill openings; no pectoral fins; snout subconical; anterior nostrils tubular and extremely short; teeth conical, mostly uniserial; gill openings ventral; and two preopercular pores (McCosker et al. 2007; McCosker 2022; this study).

### ﻿Key to species of *Yirrkala* in Taiwan

**Table:** 

1	Head with speckles	**2**
–	Head without speckles	**3**
2	Dorsal-fin origin behind gill opening by less than one head length; speckles present from head to anterior portions of trunk	***Y.misolensis* (Günther, 1872)**
–	Dorsal-fin origin far behind gill opening and slightly behind anus; speckles restricted in head	***Y.omanensis* (Norman, 1939)**
3	Dorsal-fin origin behind gill opening; lateral-line pores margined by pale spots blank at least anterior to anus in preserved condition	***Y.kaupii* (Bleeker, 1858)**
–	Dorsal-fin origin above gill opening; lateral-line pores without margin in preserved condition	***Y.nkust* sp. nov.**

### ﻿ Yirrkala nkust sp. nov.

Taxon classificationAnimaliaAnguilliformesOphichthidae

50B54A9D-F018-56E5-956A-358C9A0EC9E2

https://zoobank.org/8EAF8FC7-BEC3-41B8-9F98-3EBEADD0DBF1

**Figure 1.**
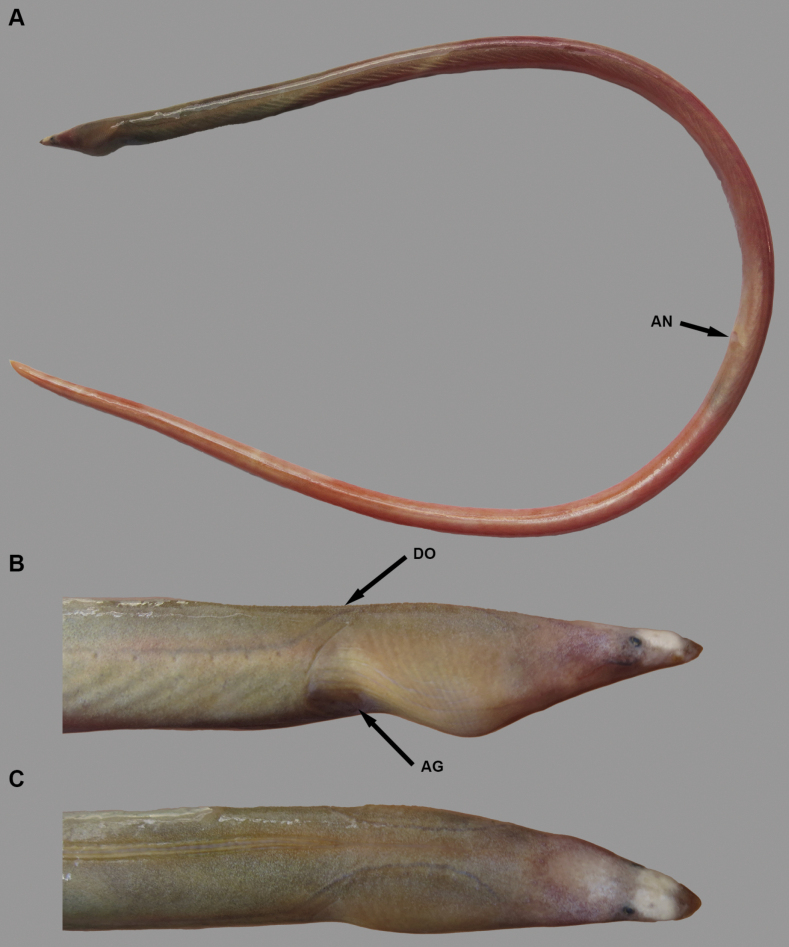
Fresh condition of *Yirrkalankust* sp. nov., holotype, NMMB-P38652, 496 mm TL**A** whole body **B** lateral view of head **C** dorsal view of head. **AG** anterior edge of gill opening **AN** anus **DO** dorsal-fin origin.

**Figure 2.**
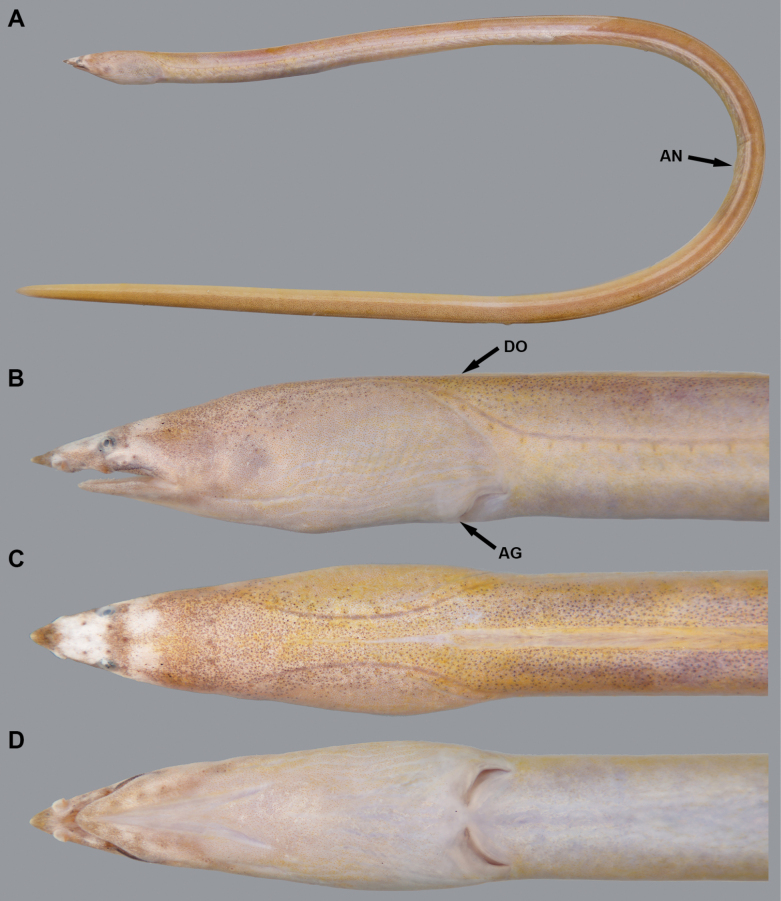
Fresh condition (after freezing) of *Yirrkalankust* sp. nov., paratype, KMNH VR 100650, 297 mm TL, photographed by Y.-C. Hsu **A** whole body **B** lateral view of head **C** dorsal view of head **D** ventral view of head. **AG** anterior edge of gill opening **AN** anus **DO** dorsal-fin origin.

**Figure 3.**
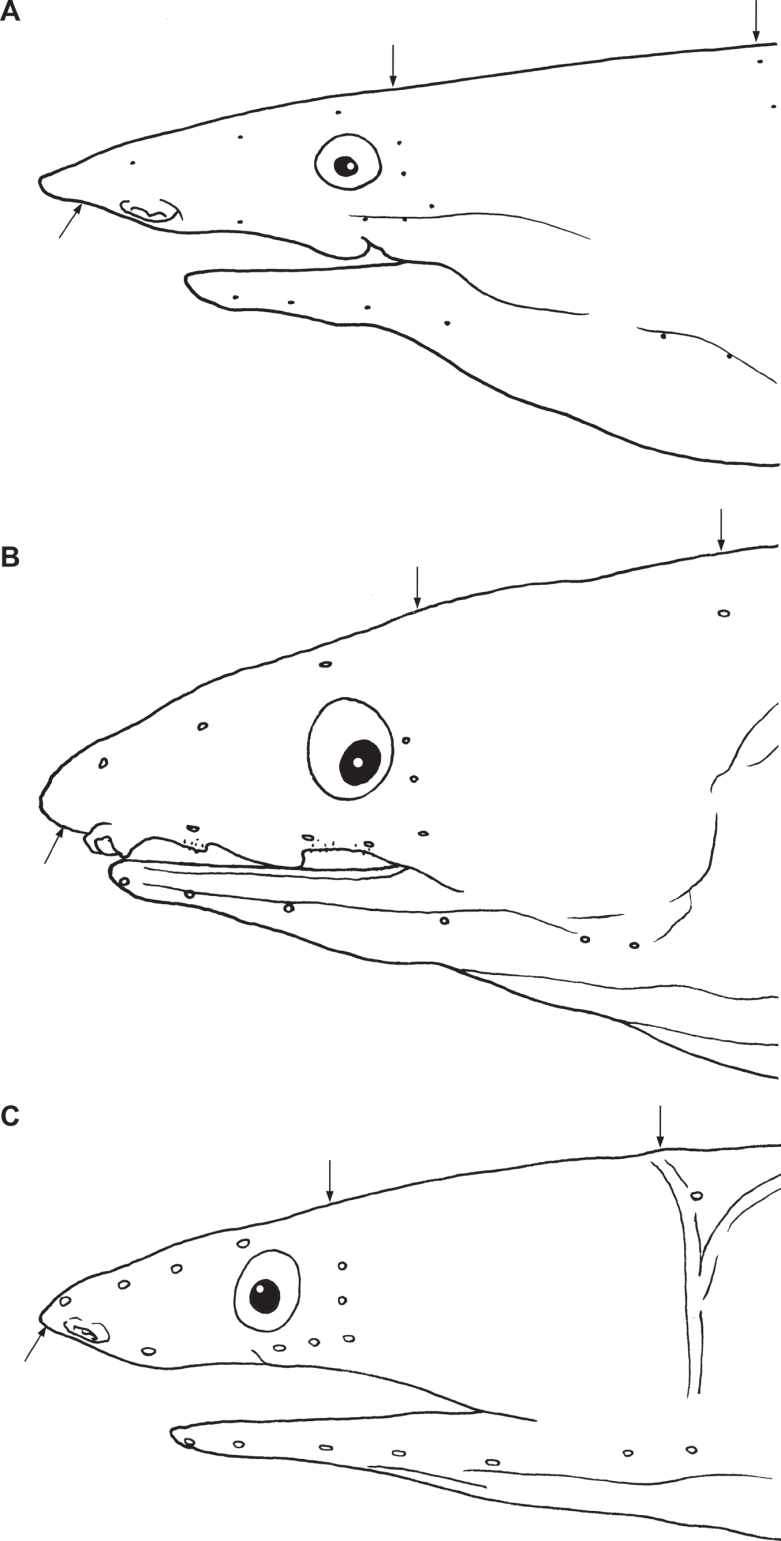
Line drawing of lateral head **A***Yirrkalankust* sp. nov., holotype, NMMB-P38652, 496 mm TL**B***Yirrkalakaupii*, NMMB-P38424, 328 mm TL**C***Yirrkalaomanensis*, KMNH VR 100616, 216 mm TL. Arrows indicate ethmoid = 1^st^ supraorbital (lefts), interorbital (middles) and mid-temporal (rights) pores.

Figs 1 , 2 , 3A , Table 1

#### Materials examined.

***Holotype*** • NMMB-P38652, 496 mm TL, ca 23°40'N, 119°36.6'E, Chi-kan, Bai-sha, northern Penghu, western Taiwan, Taiwan Strait, ca 30–50 m depth, 12 July 2021. ***Paratypes*** • NMMB-P38645, 315 mm TL, 12 July 2021; NMMB-P39317, 462 mm TL, 16 August 2022; KMNH VR 100650, 297 mm TL, 23 July 2024; all collected from near the type locality.

**Table 1.** Counts and measurements of three *Yirrkala* species newly collected from Taiwan, with type information.

	*Y.nkust* sp. nov.		*Y.kaupii*		*Y.omanensis*	
	Holotype	Paratypes	Present materials	Holotype	Present material	Holotype
Total length (mm)	496	297–462 (*n* = 3)	238–331 (*n* = 3)	342	216	230^b^
As % of TL						
Head length	5.9	6.2–6.4	7.3–8.4	6.5^a^	8.1	7.7^b^
Preanal length	51.9	47.4–52.6	47.1–47.3	N/A	57.9	58.3^c^
Tail length	48.1	47.4–52.6	52.7–52.9	N/A	42.1	41.7^c^
Predorsal length	5.1	5.5–6.1	12.5–13.5	N/A	59.8	ca 59^d^
Body depth at gill opening	1.7	1.6–1.8	2.1–2.4	(1.7)^a^	2.3	(1.8)^c^
Body width at gill opening	1.5	1.5–1.6	1.4–1.7	N/A	2.1	N/A
Body depth at midanus	1.7	1.3–1.5	2.4–2.5	N/A	2.1	N/A
Body width at midanus	1.6	1.4–1.6	1.5–2.1	N/A	2.0	N/A
As % of head length						
Snout length	15.1	14.7–16.5	13.9–14.5	ca 16^a^	11.4	ca 11^b^
Eye diameter	4.5	3.6–4.5	6.1–6.5	ca 8^a^	5.7	N/A
Upper-jaw length	28.0	28.0–30.9	22.4–23.4	ca 25^a^	28.6	N/A
Gill-opening length	12.7	11.4–15.7	8.2–13.4	N/A	9.7	N/A
Interorbital width	7.9	6.6–9.8	6.5–8.2	N/A	5.1	N/A
Isthmus width	5.1	3.9–5.5	8.5–15.6	N/A	6.9	N/A
Counts						
Predorsal vertebrae	8	7–8	17–18	17	78	76
Preanal vertebrae	72	72–75	64–66	65	76	76
Total vertebrae	147	150–152	151–155	156	141	140

Body depths in parentheses are possibly the maximum depth. ^a^from Bleeker (1858); ^b^from Norman (1939); ^c^from McCosker (2022); and ^d^estimated from Norman (1939).

#### Diagnosis.

An elongate species of *Yirrkala* with the following combination of characters: body reddish brown without speckles, snout paler; supraorbital pores (SO) 1 + 3; dorsal-fin origin just above gill opening; lateral-line pores before anus 72–78; predorsal vertebrae 7–8, preanal 72–75, and total 147–152; MVF 8-73-150.

#### Description.

Body elongate, subcylindrical, tip of tail laterally compressed and extremely pointed (Figs 1A, 2A). Head moderate in size, 15.7–17.0 in TL (17.0 in holotype); preanal length mostly equal to tail, 0.9–1.1 in tail length and 1.9–2.1 in TL (0.9 and 1.9 in holotype).

Snout relatively longer, 3.4–3.7 times eye diameter (3.7 in holotype), its tip pointed; distinct median groove ventrally on snout, its anterior tip reaching to midpoint of anterior-nostril-tube base; slope of dorsal surface of snout smooth, without notch or distinct hump. Anterior nostril tubular, short, tube length about equal to pupil diameter; posterior nostril oval in shape with an inner valve, located on anteroventral margin of eye, opening ventrally, covered by a flap extending slightly below edge of mouth gape. Eye small, covered by a transparent skin; center of eye anterior to mid-jaw (Figs 1B, 2B). Interorbital region wide, weakly convex (Figs 1C, 2C). Mouth inferior, distance from tip of snout to anterior tip of lower jaw 3.0–3.3 times eye diameter (3.3 in holotype); lower jaw short, its tip relatively pointed, not reaching anterior-nostril tube (Figs 1B, 2B, D); rictus short, posterior end of gape slightly behind a vertical through posterior margin of eye; lips smooth with a fold along upper lip, extending from second infraorbital pore to postorbital pore or rictus. Gill openings positioned ventrolaterally of breast, relatively close each side; shape of opening slightly curved, diameter more than twice eye diameter.

Sensory pores on head developed, arrangement of those pores as follows (Fig. 3A): 1 (ethmoid) + 3 on supraorbital, 3 + 3 on infraorbital, 4 on lower jaw, 2 on preopercle, and 5 on supratemporal, one of those on mid-temporal; a single median interorbital pore.

Lateral-line pores small but obvious. Lateral line almost complete except for near tip of tail, nine anterior to a vertical through gill opening, seven or eight (eight in holotype) anterior to dorsal-fin origin, 72–78 (77) anterior to mid anus, and total 149–152 (151).

Teeth pointed, slightly recurved posteriorly; 12–15 teeth on maxilla and 12–13 dentary uniserial; 10–12 vomerine teeth biserial anteriorly and uniserial posteriorly; intermaxillary with four teeth arranged in two rows.

Median fins low but obvious; origin of dorsal fin slightly behind a vertical through anterior edge of gill opening but not behind posterior edge, ending anterior to tip of tail about 1 eye diameter; origin of anal fin slightly behind anus, ending same as dorsal fin; caudal and pectoral fins absent.

***Coloration*.** Just after captured (Fig. 1), body greenish brown anteriorly, reddish brown posteriorly; lateral-line pore not margined; head generally greenish brown, postorbital region pinkish; snout with extremely pale brown transverse band dorsally in holotype, paratype also paler but tip of snout not darker; fins similar color of body. After preservation, body color faded, changed to brown to pale brown, trunk weakly bicolored, melanophores remaining dorsally.

#### Etymology.

The scientific name *nkust* is the acronym of the National Kaohsiung University of Science and Technology, Kaohsiung, Taiwan, which supports our research work. Used as a noun.

#### Distribution.

Known from the northern regions of Penghu Islands, an archipelago in the Taiwan Strait off western Taiwan. The type series was collected from bycatches of the Silver-stripe round herring (*Spratelloidesgracilis*) at depths about 30–50 m.

#### Remarks.

The position of the dorsal-fin origin compared to that of gill opening is an important character for diagnosing *Yirrkala* species. Based on the morphological information of several species (i.e. *Yirrkalaori* McCosker, 2011 and *Y.misolensis*), the origin of dorsal fin quite consistent, showing less intraspecific variation.

The new species has the dorsal-fin origin situated above gill opening (verticals between anterior and posterior edges of the opening). The character is shared by *Y.ori* only, as other 13 species, including *Y.chaselingi*, *Y.lumbricoides*, *Yirrkalamoorei* McCosker, 2006, *Yirrkalatenuis* (Günther, 1870) before; *Yirrkalacalyptra* McCosker, 2011, *Y.gjellerupi*, *Yirrkalainsolitus* McCosker, 1999, *Y.kaupii*, *Yirrkalamacrodon* (Bleeker, 1863), *Yirrkalamaculata* (Klausewitz, 1964), *Y.misolensis*, *Yirrkalaomanensis* (Norman, 1939), *Yirrkalaphilippinensis* (Herre, 1936), have their fin origins clearly behind the gill opening (Bleeker 1863; Whitley 1940; McCosker 1999, 2006; McCosker et al. 2007; McCosker 2011; Chiu et al. 2022; this study).

*Yirrkalankust* sp. nov. differs from *Y.ori* in having 1 + 3 supraorbital pores (vs 1 + 4), and the position of the lower-jaw tip (not reaching base of the anterior-nostril tube vs beyond posterior edge of the base). The new species has similar vertebral counts of *Y.lumbricoides* but not overlapped in the count of the predorsal vertebrae (7 or 8 vs 5 in *Y.lumbricoides*), and the total vertebrae (147–152 vs 150–159) is available (McCosker 2022; this study).

In addition, although *Muraenafusca* Zuiwe, 1793 was regarded as a valid species of *Yirrkala* by Fricke et al. (2018), this name should be treated as a *nomen dubium*. Its original description is insufficient which lacks direct evidence to identify it to any ophichthid and whereabouts of its holotype is unknown. *Sphagebranchusbrevirostris* Peters, 1855, which has been regarded as conspecific with *M.fusca* (Fricke et al. 2018), should be treated in a future work.

### ﻿ Yirrkala kaupii

Taxon classificationAnimaliaAnguilliformesOphichthidae

(Bleeker, 1858)

97FDEBB4-6C8C-531E-9B1D-47FDB09E671F

**Figure 4.**
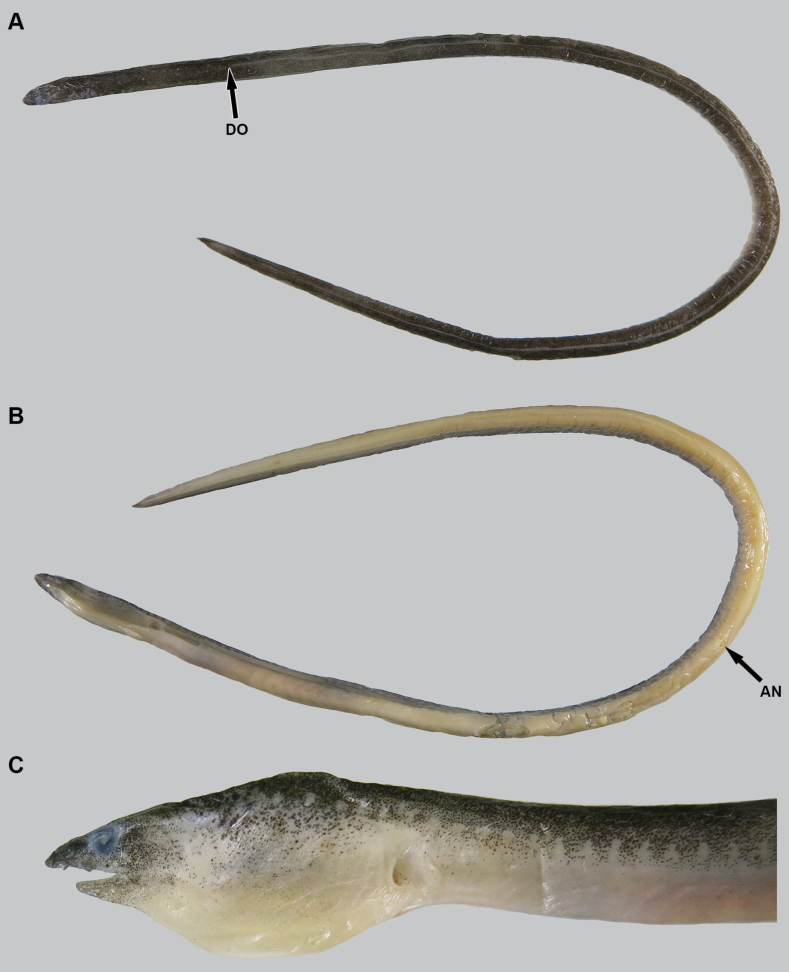
Preserved specimen of *Yirrkalakaupii*, NMMB-P36108, 239 mm TL**A** whole body (dorsal view) **B** whole body (ventral view) **C** enlarged view of head (lateral). **AN** anus **DO** dorsal-fin origin.

Figs 3B , 4 , Table 1

Sphagebranchus kaupii Bleeker, 1858: 3 (type locality: Manado, Sulawesi, Indonesia); Bleeker 1864: 70 (Manado).
Ophichthys kaupi : Günther 1870: 86 (Celebes).
Sphagebranchus kaupi : Weber and de Beaufort 1916: 325 (Celebes).
?Yirrkalakaupi: McCosker 1977: 16 (listed; specimen collected from Philippines); McCosker 2014: 339 (listed).
Yirrkala kaupii : Kottelat et al. 1993:13 (listed, Indonesia); Smith and McCosker 1999: 1669 (listed); Miesen et al. 2016: 81 (listed, Indonesia); Jamandre 2023: 160 (listed, Indonesia).

#### Materials examined.

• NMMB-P36108, 239 mm TL, ca 23°56.2'N, 121°36.5'E, jointed mouth of Mu-Gua River and Hualien River, Hualien, eastern Taiwan, ca 1 m, trap net (fyke net), 10 May 2021 • NMMB-P38424, 328 mm TL, NMMB-P38425, 331 mm TL, ca 23°27.9'N, 121°29.7'E, Jin-pu village, Fengbin township, Taitung, 26 May, 2023, collected after preserving in wine for several years.

#### Diagnosis.

An elongate species of *Yirrkala* with the following combination of characters: body bicolored, dark brown dorsally and pale ventrally, lateral line pores margined as pale blank; SO 1 + 3; dorsal-fin origin behind gill opening; lateral-line pores before anus 63–65; predorsal vertebrae 17–18, preanal 64–66, and total 151–156; MVF 17-65-154.

#### Description of Taiwanese specimens.

New record. Body elongate, subcylindrical, tip of tail laterally compressed and extremely pointed (Fig. 4A, B). Head moderate in size, 11.8–13.7 in TL; preanal length shorter than tail, 1.1 in tail length and 2.1 in TL.

Snout relatively longer, more than twice eye diameter, its tip pointed; distinct median groove ventrally on snout but the groove mostly not teared, its anterior tip beyond ethmoid pore; slope of dorsal surface of snout smooth, without notch or hump. Anterior nostril tubular, moderate in length, tube length about equal to pupil diameter; posterior nostril oval in shape with an inner valve, located on anteroventral margin of eye, opening ventrally, covered by a flap extending slightly below edge of mouth gape. Eye moderate in size, covered by a transparent skin; center of eye anterior to mid-jaw (Fig. 4C). Mouth inferior, distance from tip of snout to anterior tip of lower jaw slightly longer than eye diameter; lower jaw short, its tip relatively pointed, slightly beyond or reaching posterior base of anterior-nostril tube (Fig. 4C); rictus short, posterior end of gape slightly behind a vertical through posterior margin of eye; lips smooth with folds, the fold along upper lip extending from second infraorbital pore to posterior rictus, but absent in one specimen (NMMB-P38425); the fold along lower lip extending from position between first and second mandibular pores to anterior of first preopercular pore. Gill openings positioned ventrolaterally of breast, not close each side; shape of opening curved, diameter more than twice eye diameter.

Sensory pores on head developed, arrangement of those pores as follows (Fig. 3B): 1 (ethmoid) + 3 on supraorbital, 3 + 3 on infraorbital, 4 or 5 on lower jaw, 2 on preopercle, and 3 on supratemporal, one of those on mid-temporal; a single median interorbital pore.

Lateral-line pores small but obvious. Lateral line almost complete except for near tip of tail, eight or nine anterior to a vertical through gill opening, 16–18 anterior to dorsal-fin origin, 63–65 anterior to mid anus, and total 116–138.

Teeth pointed, slightly recurved posteriorly; teeth on maxilla, vomer and dentary uniserial; maxilla comprising 19 (right)/25 (left) teeth and mandible comprising 21/23 teeth in NMMB-P36108; intermaxillary with four or five teeth arranged in two rows or a chevron shape.

Median fins low but obvious; origin of dorsal fin behind gill opening, ending anterior to tip of tail about 1 eye diameter; origin of anal fin slightly behind anus, ending same as dorsal fin; caudal and pectoral fins absent.

***Coloration*.** No information of fresh coloration. After the preservation by alcohol (ca 60%) directly, body clearly bicolored, generally darkish brown dorsally and pale yellowish brown ventrally; numerous melanophores present dorsally, the border going down toward tip of tail; lateral-line pores margined by pale spots blank at least anterior to anus, but in tail the pattern gradually faded; head darker dorsally, lower jaw also dusky; dorsal fin dusky but margin pale yellowish white; eye with whitish margin by skin covering eye; anal fin pale yellowish white except dusky tip of tail ca 1/2 HL.

#### Distribution.

Manado, Sulawesi, Indonesia (holotype) and eastern Taiwan. In both places, this species was collected from rivers, but there is no detailed information for the holotype.

#### Ecological note.

One specimen was collected from a river mouth together with many *Lamnostoma* spp., a genus which is commonly found in the freshwater environments. However, the species might be rare because only one individual was found among approximately 200 individuals of *Lamnostoma*.

#### Remarks.

**Figure 5.**
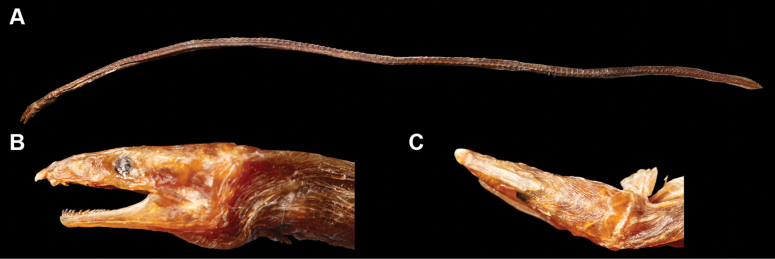
Holotype of *Sphagebranchuskaupii*, BMNH 1867.11.28.304, 342 mm TL**A** whole body **B** lateral view of head **C** dorsal view of head. All photos provided by Natural History Museum.

*Sphagebranchuskaupii* is one of the oldest names in the genus *Yirrkala*, and it lacks detailed morphological information except for the original description (Bleeker 1858). Based on YH’s investigation, the only known Bleeker specimen, deposited in the Natural History Museum (BMNH 1867.11.28.304), should be regarded as the holotype because its similar length agrees with the original description (e.g. 342 mm TL vs 362 mm TL in the original description) (Fig. 5).

Our specimens closely match the holotype in body proportions and vertebral counts in the original description. Bleeker (1858) noted that it has drop-shaped markings on the lateral line, which are also found in our specimens. Although Günther (1870) mentioned that it has uniform body coloration, it is assumed that holotype specimen was once dried and bleached beforehand because its very poor present condition had been caused by the drying process. There are several differences in the proportions of the head, snout, eye, and upper-jaw lengths between the original description of *S.kaupii* and Taiwanese specimens (Table 1). The Taiwanese population is possibly an undescribed species, but the morphological differences could also be due to intraspecific variation or differences in the method of preservation, or the time in preservative. As we were unable to directly observe the holotype and the number of specimens observed was limited, we refrain from considering it a separate species.

Most of congeners of *Yirrkala* have a dorsal fin that originates around, and usually slightly behind, the gill opening (see above). Only four species, *Y.gjellerupi*, *Y.kaupii*, *Y.insolitus*, and *Y.omanensis*, have the origin of the fin located more than half a head length behind the gill opening. Moreover, the latter two species are unique because their dorsal-fin origins are situated far behind, near the anus. *Yirrkalakaupii* is most similar to *Y.gjellerupi* in the vertebral counts and body proportions, including head length, tail length, and snout length. Both species inhabit rivers far from the river mouth (McCosker et al. 2007). However, *Y.kaupii* can be distinguished from *Y.gjellerupi* by the position of the rictus (behind a vertical through posterior margin of eye, vs before), and the presence of a small protrusion along around margin of posterior nostril (vs absent).

### ﻿ Yirrkala misolensis

Taxon classificationAnimaliaAnguilliformesOphichthidae

(Günther, 1872)

C0B770A1-1C2D-5D46-AD1B-A629C127C644

Ophichthys misolensis Günther, 1872:426 (type locality: Misool [Misol] Island, Irian Jaya, Indonesia).
Dalophis misolensis : Jordan and Seale 1906: 194 (Misol).
Yirrkala misolensis : McCosker 1977: 69 (listed); Smith and McCosker 1999: 1669 (listed); McCosker et al. 2006: 277 (listed, Queensland, Australia); Menes et al. 2010: 98 (Bago River, Negros and Panay, Philippines); McCosker 2014: 339 (listed, Philippines); Ho et al. 2015: 177 (Taiwan, listed as a new record); Motomura et al. 2017: 35 (Panay, Philippines); Hibino 2019: 154 (Dong-gang, Taiwan); Hibino et al. 2021: 21 (Okinawa, Japan); Chiu et al. 2022: 117 (Taiwan, redescription); McCosker 2022: 136 (Maldives, but photographed specimen collected from Taiwan).

#### Diagnosis.

An elongate species of *Yirrkala* with the following combination of characters: body pale to dark brown with mottled patterns from snout to anterior trunk; SO 1 + 3; dorsal-fin origin behind gill opening; lateral-line pores before anus 76–85; total vertebrae 165–180, MVF 10-77-173 (Hibino et al. 2021; Chiu et al. 2022).

#### Distribution.

Indo-Pacific from India to Fiji, including Indonesia (holotype), north to Ryukyu Islands, Japan; specimens collected from Dong-gang, Ke-tzu-liao, southwestern part of Taiwan (Chiu et al. 2022).

#### Remarks.

**Figure 6.**
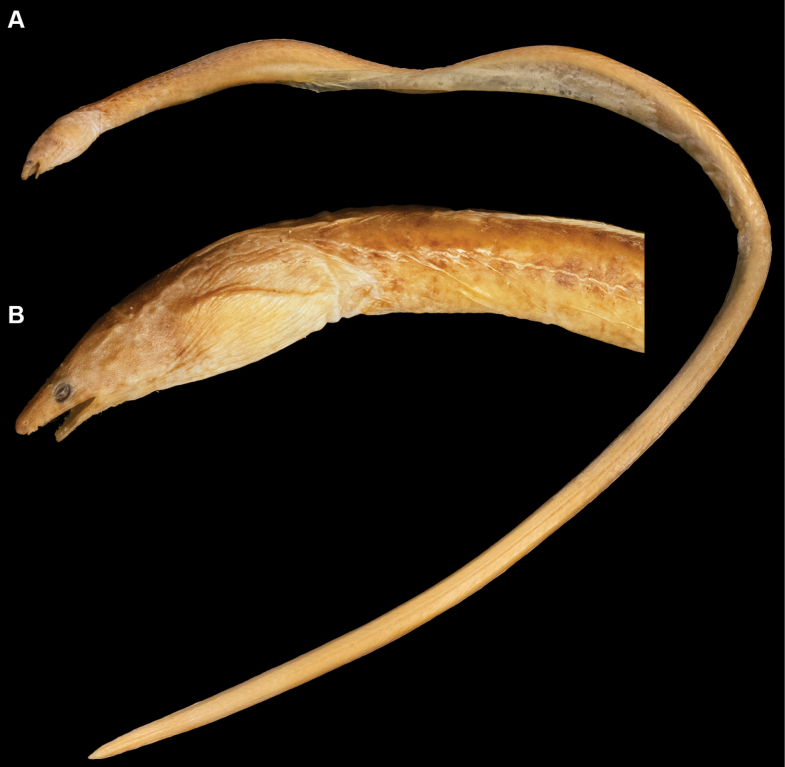
Holotype of *Ophichthysmisolensis*, BMNH 1870.8.31.112, 280 mm TL**A** whole body **B** head and breast.

Ho et al. (2015) first listed this species from Taiwan with voucher specimens. Chiu et al. (2022) provided a detailed redescription and the results of DNA barcoding. It is notable that McCosker (2022) provided the MVF 12-75-168 and total vertebrae 166–169 which is slightly different from our observation. It is likely that the *Y.misolensis* from Taiwan may include different populations. Without an explanation, McCosker (2022) regarded *Caeculamaculata* Klausewitz, 1964 (type locality: Nicobar Islands) as a junior synonym of *Y.misolensis*. However, we cannot make any judgment about this synonymy without examining specimens from the type locality of *C.maculata*. The correct catalog number of the holotype of *Ophichthysmisolensis* is BMNH 1870.8.31.112 (https://data.nhm.ac.uk/dataset/56e711e6-c847-4f99-915a-6894bb5c5dea/resource/05ff2255-c38a-40c9-b657-4ccb55ab2feb/record/3103514), although Hibino et al. (2021) and Chiu et al. (2022) erred and gave the wrong catalogue number (Fig. 6).

### ﻿ Yirrkala omanensis

Taxon classificationAnimaliaAnguilliformesOphichthidae

(Norman, 1939)

0D28D121-9461-58B3-ABDC-EB5F232A0A8E

**Figure 7.**
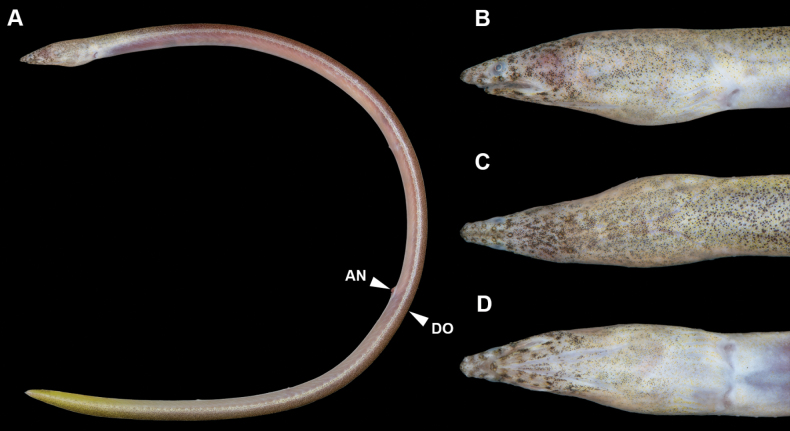
Fresh condition of *Yirrkalaomanensis*, KMNH VR 100616, 216 mm TL, photographed by H. Kobayashi **A** whole body **B** lateral view of head **C** dorsal view of head **D** ventral view of head. **AN** anus **DO** dorsal-fin origin.

Figs 3C , 7 , Table 1

Sphagebranchus omanensis Norman, 1939 (type locality: Gulf of Oman).
Ichthyapus omanensis : Randall 1995: 61 (Gulf of Oman); Manilo and Bogorodsky 2003: S95 (list, coast of Oman).
Yirrkala omanensis : McCosker 2022: 136 (Gulf of Oman).

#### Material examined.

• KMNH VR 100616, 216 mm TL, Ke-tzu-liao, Kaohsiung, southwestern Taiwan, 6 March 2024, collected H. Kobayashi and Y. Hibino.

#### Diagnosis.

A relatively elongate species of *Yirrkala* with the following combination of characters: body reddish brown mostly except yellow tail end, with speckled patterns on head; SO 1 + 4; dorsal-fin origin well behind gill opening, slightly behind anus; lateral-line pores before anus 77; total vertebrae 140–141, MVF 77-76-141.

#### Description based on KMNH VR 100616.

New record. Counts and measurements are shown in Table 1. Body elongate, cylindrical, tip of tail laterally compressed and extremely pointed (Fig. 7A). Head moderate in size, 12.3 in TL; preanal length much longer than tail, 0.7 in tail length and 1.7 in TL.

Snout moderate in length, twice eye diameter, its tip weakly pointed; distinct median groove ventrally on snout but the groove opened completely, its anterior tip beyond anteriormost margin of first infraorbital pore; slope of dorsal surface of snout smooth, without notch or hump. Anterior nostril tubular but extremely short, tube length about half of pupil diameter; posterior nostril oval in shape with an inner valve, located on anteroventral margin of eye, opening ventrally. Eye moderate in size, covered by a transparent skin; center of eye anterior to mid-jaw (Fig. 7B). Interorbital region relatively narrow, weakly convex with an extremely shallow dimple (Fig. 7C). Mouth inferior, distance from tip of snout to anterior tip of lower jaw slightly shorter than twice eye diameter; lower jaw short, its tip relatively pointed and located anterior to a vertical through anterior margin of eye (Fig. 7B, D); rictus short, posterior end of gape well behind a vertical through posterior margin of eye; lips smooth with folds, the fold along upper lip weak, extending from a vertical through second infraorbital pore to posterior rictus; the fold along lower jaw short but obvious, extending from fourth mandibular pore to just before of first preopercular pore. Gill openings positioned ventrolaterally of breast close but not connected each side; shape of opening curved, diameter less than twice eye diameter.

Sensory pores on head developed, arrangement of those pores as follows (Fig. 3C): 1 (ethmoid) + 4 on supraorbital, 3 + 3 on infraorbital, 5 on lower jaw, 2 on preopercle, and 3 on supratemporal, one of those on mid-temporal; a single median interorbital pore. Lateral-line pores small but obvious. Lateral line almost complete except for near tip of tail, 8 anterior to a vertical through gill opening, 77 anterior to mid anus, 79 anterior to dorsal-fin origin, and total 136.

Teeth pointed, slightly recurved posteriorly; teeth on maxilla and dentary uniserial; teeth on vomer biserial irregularly anterior one-third and remaining uniserial; intermaxillary teeth slightly larger and more slender than maxillary teeth, three teeth arranged in a chevron shape along with edge of pre-ethmoid; intermaxillary teeth visible when mouth is closed.

Median fins very tiny and rudimental; origin of dorsal fin slightly behind that of anal fin, ending anterior to tip of tail about one snout length; origin of anal fin slightly behind anus, ending same as dorsal fin; caudal and pectoral fins absent.

***Coloration*.** Just after captured (Fig. 7), body generally reddish brown, gradually changing to yellowish brown to brilliant yellow in posterior tail; numerous tiny melanophores covering dorsolateral body, ventral side without melanophores except tip of tail; numerous xanthophores also present including ventral side. Base color of head pale brown, coloring weaker than body; head with both melanophores and xanthophores, the former gathering and making speckles, arranged as a row along lips and postorbital region; pores on snout prominently margined, but others not margined necessarily, some connected with speckle. Dorsal and anal fins semitransparent white without darker margin. After preservation, brilliant yellow and red completely faded but all speckled remaining; transparent skin on eye becoming whitish.

#### Distribution.

Gulf of Oman (holotype) and southwestern Taiwan (this study). The Taiwanese specimen was estimated to be collected from a depth of around 10–50 m, based on the bycatches collected in the same haul.

#### Remarks.

**Figure 8.**
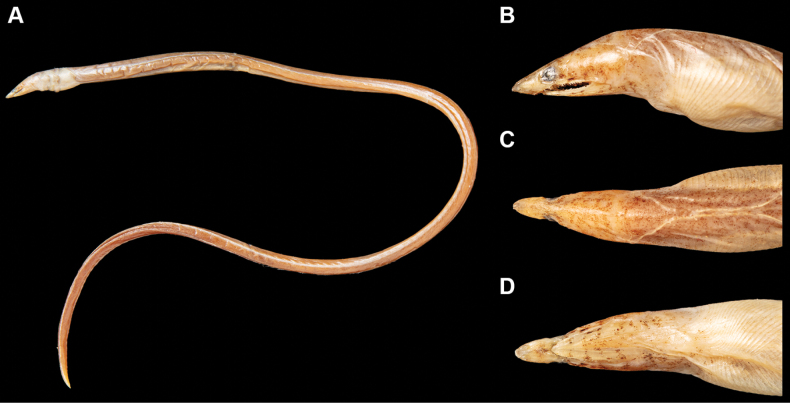
Holotype of *Sphagebranchusomanensis*, BMNH 1939.5.24.650, 230 mm TL**A** whole body **B** lateral view of head **C** dorsal view of head **D** ventral view of head. All photos provided by Natural History Museum.

Our specimen agrees well with the holotype in counts and body proportions (Table 1), as well as in the diagnostic feature of the speckles on head (Figs 7, 8). The present specimen represents the second known specimen of *Y.omanensis* and the first record from the Pacific Ocean. This may suggest that *Y.omanensis* has a wide distribution in the Indo-West Pacific Ocean. However, additional specimens are needed to fully understand the complete distribution range of the species.

*Yirrkalaomanensis*, originally *Sphagebranchusomanensis*, was described by Norman (1939) with a simple line drawing. McCosker (1977) suggested it is an *incertae sedis*, but subsequently, McCosker (2011) included it in *Yirrkala* without further explanation. Notably, this species is very unique in having the dorsal-fin origin situated far posterior, behind the anus, compared to a more anterior origin near the gill opening, such as in all other species of the genus except *Y.insolitus*. However, we agree with the McCosker’s (2011) taxonomic placement of this species in *Yirrkala* because all other characters of *Y.omanensis* are consistent with this genus.

#### Comparative materials.

*Yirrkalakaupii*: BMNH 1867.11.28.304 (holotype), 342 mm TL, Manado, Sulawesi, Indonesia. *Yirrkalalumbricoides*: BMNH 1867.11.28.322 (holotype), 229 mm TL, Timor, southern Malay Archipelago. *Yirrkalamisolensis*: BMNH 1870.8.31.112 (holotype), 280 mm TL, Misol Island, Irian Jaya, Indonesia. *Yirrkalaomanensis*: BMNH 1939.5.24.650 (holotype), 230 mm TL, Gulf of Oman. *Yirrkalaphilippinensis*: SU (CAS) 30977 (holotype), 365 mm TL, Dumaguete, Oriental Negros, Philippines. *Yirrkalatenuis*: BMNH 1965.1.2.1 (lectotype), no locality.

## Supplementary Material

XML Treatment for Yirrkala

XML Treatment for Yirrkala nkust

XML Treatment for Yirrkala kaupii

XML Treatment for Yirrkala misolensis

XML Treatment for Yirrkala omanensis

## ﻿Additional information

### Author ORCIDs

Yusuke Hibino https://orcid.org/0000-0002-5670-3851

Hsuan-Ching Ho https://orcid.org/0000-0003-1154-601X

### Data availability

All of the data that support the findings of this study are available in the main text.
